# AQP4 knockout promotes neurite outgrowth via upregulating GAP43 expression in infant rats with hypoxic‐ischemic brain injury

**DOI:** 10.1002/ibra.12062

**Published:** 2022-08-19

**Authors:** Qi‐Qin Dan, Zheng Ma, Ya‐Xin Tan, Belegu Visar, Li Chen

**Affiliations:** ^1^ National‐Local Joint Engineering Research Center of Translational Medicine of Anesthesiology, West China Hospital Sichuan University Chengdu China; ^2^ Center for Epigenetics and Induced Pluripotent Stem Cells, Kennedy Krieger Institute Johns Hopkins University Baltimore USA

**Keywords:** AQP4, axonal growth, behavioral improvement, GAP43, neonatal hypoxic‐ischemic encephalopathy

## Abstract

Neonatal hypoxic‐ischemic encephalopathy (NHIE) induces severe cerebral damage and neurological dysfunction, with seldom effective therapy. Aquaporin‐4 (AQP4) is involved in aggravating brain damage induced by NHIE. This study aimed to investigate the role of AQP4 underlying the pathogenesis of NHIE. Neonatal Sprague–Dawley rats were used to establish neonatal hypoxic‐ischemic (HI) models, and the expression of AQP4 in the cortex, hippocampus, and lung tissues was detected by real‐time quantitative polymerase chain reaction as well as Western blot. Primary cortical neurons were cultured for the oxygen‐glucose deprivation (OGD) model, and siRNA was used to silence the expression of AQP4. Immunostaining of Tuj1 was performed to observe the axonal growth. CRISPER/Cas9 technology was used to knock out AQP4. The results demonstrated that AQP4 was upregulated in the cortex, hippocampus, and lung tissues in neonatal rats with HI and OGD neurons. Besides, silencing AQP4 promoted axonal growth of OGD neurons, and AQP4 knockout notably improved long‐term neurobehavioral impairment. Furthermore, GAP43 was found closely correlated with AQP4 via GeneMANIA prediction. Significant downregulation of GAP43 was induced in OGD neurons, while AQP4 knockout markedly upregulated its expression in rats. This indicated that the depletion of AQP4 may enhance axonal regeneration and promote the long‐term neurobehavioral recovery associated with the upregulation of GAP43 expression.

## Graphical abstract

Neonatal Sprague–Dawley rats (7 days old) were applied to establish hypoxic‐ischemic (HI) models; moreover, siRNA and CRISPER‐/Cas9‐mediated gene‐editing technologies were then employed to explore the role of AQP4 in the neurological damages at 24 h after HI in vitro and in vivo. The results revealed that silencing AQP4 could promote the outgrowth of the length in oxygen‐glucose deprivation (OGD) neurons. Moreover, AQP4‐knockout could notably improve long‐term neurobehavioral impairment compared with AQP4‐wild type 1 month after HI reflected by the tests of neurological severity score, water maze, and Y‐maze. Furthermore, growth‐associated protein‐43 (GAP43) was closely correlated with AQP4 in both pathway and co‐expression channels via GeneMANIA prediction. As expected, OGD induced significant downregulation of GAP43, while AQP4 knockout could markedly upregulate its expression. These indicated that AQP4 silencing could enhance axon regeneration and promote long‐term neurobehavioral recovery after HI; the underlying mechanism was associated with GAP43 upregulation.

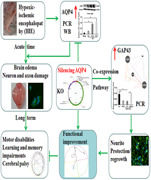

## 1 INTRODUCTION

Neonatal hypoxic‐ischemic encephalopathy (NHIE) is a common cerebral injury triggered by asphyxia during birth.^1^, ^2^ An outsized proportion of patients with NHIE suffer severe long‐term cognitive and motor impairment, for instance, general developmental delay, cerebral palsy, epilepsy, learning difficulties and irreversible brain damage, and so forth, resulting in low life quality in patients and economic drawbacks in society, particularly in low‐ and middle‐income countries.^3^, ^4^, ^5^, ^6^, ^7^ The pathogenesis of NHIE is complex, of which the primary phase exhibits changed vasculature, and involves a primary energy failure section occurring at the cellular level.^8^, ^9^ The secondary phase was thought to incorporate mechanisms of inflammation, impairment or alteration of nerve fiber growth, development, and synaptogenesis, of which the dysfunction of axons in the nervous system could affect both peripheral and central neurons.^10^, ^11^ Furthermore, during the acute phase, continued injury cascade like cytotoxicity, oxidative stress, mitochondrial disorder, and autophagy in the nerve cells or connection would cause long‐term neurological dysfunction.^12^ All these factors could contribute to final outcomes of neuronal damages. Therefore, promoting axonal regeneration could be therapeutically targeted for the treatment of NHIE.

Aquaporin‐4 (AQP4), a water channel supermolecule encoded by the AQP4 factor in humans, is localized on the plasma membrane of skeletal muscle, as well as kidney, lung, stomach, and brain, taking a vital part in the formation of brain edema.^13^, ^14^, ^15^, ^16^, ^17^, ^18^ It is commonly believed that AQP4 is primarily distributed on astrocytes. However, there was evidence indicating that AQP4 can be selectively distributed on nerve cells, mainly on the nucleus or the cell body of the nerve cell layer where the cell body is relatively concentrated.^19^, ^20^ Moreover, AQP4 distributed in the hippocampus, cerebellum, brainstem, and some cortical neurons is mainly involved in the regulation of extracellular space size and K^+^ concentration in extracellular space.^21^, ^22^ Some studies showed that increased intracranial pressure following brain injury, caused by the development of brain edema, contributed to poor outcomes.^23^ It has been reported that targeting AQP4 would result in reduced brain edema and alleviated brain ischemia in which AQP4 knockdown could reduce the cerebellar edema formation, thereby alleviating the acute hypoxic‐ischemic brain injury.^24^, ^25^ It has also been reported that curcumin can decrease brain edema by protecting the blood–brain barrier (BBB) ultrastructure to downregulate the expression of AQP4 protein in HI brain damage.^26^, ^27^ However, the role and regulatory mechanism of AQP4 in the long‐term complications induced by NHIE and the role of axonal regeneration during NHIE recovery remain elusive.

## 2 EXPERIMENTAL PROCEDURES

### 2.1 Animals and grouping

Both the male and female baby Sprague–Dawley (SD) rats (7 days old, 12–15 g) were purchased from the Experimental Animal Center of Kunming Medical University. Animals were housed in individual cages with controlled temperature (21–25°C), humidity (45%–50%), and light. They have free access to food and water. AQP4^−/−^ rats were created in Cyagen Biosciences (Cyagen) using CRISPR/Cas engineering technology. This study was approved by the Animal Care and Use Committee of Kunming Medical University (kmmu2019039). All the operations were carried out in conformity to Care and Use of Laboratory Animals publicized by the Ministry of Science and Technology of the People's Republic of China and were in compliance with the Guide for the Care and Use of Laboratory Animals published by the National Institutes of Health.

### 2.2 Establishment of NHIE rat models

NHIE models were established as previously described.^28^ Briefly, all postnatal Day 7 (P7) rats divided into sham and HI groups were deeply anesthetized by inhalation of isoflurane for 3–5 minutes (min). A 0.5 cm skin incision was made at the midplane of the neck, and the right carotid artery was exposed and ligated with an electrocoagulator (Spring Medical Beauty instrumentality Co., Ltd.) at room temperature. Following the operation, the infant rats were returned to their dams to recover for 1 hour (h) and then were placed into an airtight chamber maintaining 8% O_2_ and 92% N_2_ at 37°C for 2 h. The sham rats underwent the same procedures except for the ligation of the carotid artery.

### 2.3 Wet and dry weight analysis

Wet and dry weight analysis was performed as previously described.^29^ After the experimental rats were deeply anesthetized with inhalation of isoflurane they were killed. Next, the whole brain was removed 24 h after the HI insult. The ipsilateral and contralateral cortices were collected from the pups. Later on, once cortices were weighed on aluminum foil (wet weight), they were then dried at 150°C for 12 h. The dehydrated tissue was then reweighed (dry weight). Therefore, water content was calculated by the subsequent formula: water content (%) = [(wet weight − dry weight)/wet weight] × 100. The sham rats underwent the same procedures.

### 2.4 Genotype identification

The tail tips were collected from 7‐day‐old rats, and rats' genomic desoxyribonucleic acid was extracted with Transgen's genomic desoxyribonucleic acid extraction kit (ee101‐12). Additionally, the reaction was performed in a mixtures system, including 10 µl PCR master mix, 0.6 µl upstream primers, 0.6 µl downstream primers, 3 µl DNA template, and 5.8 µl water. DNA sequencing primers for AQP4 were as follows: Rat AQP4‐F: 5′‐TCTAGGGACAGTTCAGATTACCGTCCAG‐3′, Rat AQP4‐R: 5′‐ATGTGCCACCAAACCTCACACATG‐3′. PCR amplification was executed with the procedure: initial denaturation at 94°C for 5 min, 35 cycles of denaturation at 94°C for 30 s, hardening at 59°C for 30 s, with elongation at 72°C for 30 s, followed by elongation at 72°C for 5 min. Afterwards, genotype detection was conducted using an agarose gel electrophoresis system.

### 2.5 Morris water maze test

Morris water maze test was performed to observe the spatial learning and memory dysfunction 1 month after HI injury. Briefly, the pool was divided equally into four quadrants, with the platform placed within the center of one of the quadrants. We added ink into a pool full of water until the platform was not visible in the pool. Each rat was trained for 5 days (90 s per day) in a row, and the time when the animal found the platforms were recorded. During training, if the rats failed to reach a platform in 90 s, they should be guided for target‐hunting to the platform. On the sixth day of the trial, the platform was removed for exploration coaching within 90 s. Afterward, the animals were placed within the pool from the alternative facet of the first platform quadrant. Then we recorded the time rats spent crossing the target quadrant where the platform was originally placed as the measure of spatial memory. The interval between training was 15–20 min. The animals were dried and returned to their cages after the test had finished.

### 2.6 Y‐maze test

Y‐maze test was performed to evaluate the memory function of rats. To inspire the desire of rats to ingest food, rats ought to fast for 1–2 days but water was supplied until body weight was reduced to 85%. Then, they were placed in the Y‐maze arms with 10 min residence in each arm. Subsequently, the rats were placed in one arm of the Y‐maze, and the door of the opposite arm was closed. Next, rats were located in the first section, trained to seek food, and guided if necessary. This training lasted for 5–10 min each time. Afterward, three arms of the Y‐maze doors were opened. The rats were placed into the initial arm without food. We recorded the times of rats entering each arm and the time of staying within 5 min, which was calculable by SuperMaze V2.0.

### 2.7 Neurological severity score (NSS score)

The neurological functions of rats after HI injury were evaluated by NSS scoring, which is a combination of sensory, motor, balance testing, and reflex as previously described.^30^ Neurological functions were evaluated by scores ranging from 0 to 18 (normal score: 0; maximum defect score: 18).

### 2.8 Tissue harvest

After behavioral evaluations had finished, rats in the sham and HI groups were killed by inhalation of 5% CO_2_ and then perfused with normal saline. Then the skull and thoracic cavity were opened to harvest the brain and lung tissues. Tissues used for real‐time quantitative polymerase chain reaction (RT‐qPCR) or Western blot (WB) detection were frozen at −80°C.

### 2.9 Culture of primary cortical neurons and PC12 cells

One‐day‐old SD rats were used for isolation of cortical neurons. The cortices were obtained, minced, and isolated with 0.25% trypsinase for 10 min at 37°C. Afterward, the tissues were eluted with BSA. The neurons were collected by a natural process and resuspended by 100% BSA, then covered in six‐well plates (Corning) at the density of 5 × 10^5^ cells/ml at 37°C with 5% CO_2_. Moreover, for technique staining, neurons were incubated at 37°C with five‐hitter dioxide for 4 h, and then the whole substance was modified by neurobasal medium with the addition of B27 (Invitrogen). The medium was replaced every 3 days. The PC12 cell lines were obtained from GeneCopoeia company and cultivated in Dulbecco's modified Eagle's medium (DMEM; Gibco) supplemented with fatal bovine liquid (Gibco), and placed in a very wet brooder containing 5% CO_2_ at 37°C.

### 2.10 Screening for the effective fragment of AQP4 siRNA

At first, the factor information of AQP4 was gathered from NCBI. Then, three siRNAs specifically inhibited AQP4 and one nonsense siRNA as a negative management area unit of measurement designed and purchased from Ribobio Company. Target ribonucleic acid series of fragment one (F1) is CCAAGTCCGTCTTCTACAT; fragment two (F2): CAGGTGCACTTTACGAGTA; fragment three (F3): CAGCATGAATCCAGCTCGA. In brief, once the cells reached 80% confluence, contemporary average holding siRNA portions were inserted into cells in accordance with the manufacturer's protocol from Ribobio Company. The PC12 cells were haphazardly divided into the normal group, NC group, chemical agent group, F1 group, F2 group, and F3 group. RT‐qPCR was performed to observe the consequences of siRNAs, and the most effective siRNA fragment was used for the later experiment.

### 2.11 Oxygen‐glucose deprivation (OGD) in neurons

Cortical neurons were cultured for 5 days, and then siRNA was transfected into neurons in the AQP4‐si group. Two days after transfection, the cells were subjected to OGD. In short, the neuron‐specific medium was replaced by glucose‐free DMEM (Gibco) and the neurons were cultured in a gas mixture composed of 5% CO_2_ and 95% N_2_ at 37°C for 1 h.

### 2.12 RT‐qPCR

Tissue samples were taken from the −80°C freezer for total RNA extraction using the TRIzol method. Then, they were transcribed reversely into cDNA. After that, PCR was performed with the reaction system composed of 10 µl 2 × PCR master mix, 0.6 µl forward primer, 0.6 µl reverse primer, 1 µl cDNA, and 7.8 µl PCR nuclease‐free water. The primers for detected genes were as follows:

AQP4 forward: 5’‐TCTAGGGACAGTTCAGATTACCGTCCAG‐3’;
AQP4 reverse: 5’‐ATGTGCCACCAAACCTCACACATG‐3’;
GAP43 forward: 5’‐TGTTGCCGATGGGGTGGAGA‐3’;
GAP43 reverse: 5’‐CCGTTGGAGGCTGGGCTGTT‐3’.

PCR cycling conditions were set as initial denaturation at 95°C for 5 min, 40 cycles of denaturation at 94°C for 30 s, and annealing at 59°C (AQP4), 54°C (GAP43) for 30 s, followed by elongation at 72°C for 20 s. β‐actin was used as an internal control to normalize ribonucleic acid content for each sample. The relative expression was calculated by the $2^{-\Delta \Delta C_{t}}$ method.

### 2.13 WB detection

Protein was extracted from the right cortex, right hippocampus, and lung by using RIPA buffer in an ice bath at 4°C. Protein concentration was quantitated with bicinchoninic acid protein assay. Equal amounts of samples were separated by agarose gel electrophoresis, followed by a transferring onto polyvinylidene difluoride microporous membrane. Subsequently, the membranes were plugged with Tris‐HCl buffer saline (TBS) and TBS with Tween 20 (TBST) for 2 h at room temperature, then incubated overnight at 4°C with primary antibodies of AQP4 (mouse, 1:500; Abcam). Then, the blots were washed three times with TBST for 5 min each time and incubated with secondary antibodies of AQP4 (HRP, anti‐mouse IgG, 1:5000; Abbkine) for 2 h. After washing, the immunoblot was disclosed with diaminobenzidine 3,3 staining. β‐actin was used for loading control. Finally, measuring analysis was performed with Image J software, and the relative content for AQP4 is presented by the ratio of the gray value of the target strip to the gray value of β‐actin.

### 2.14 Immunofluorescent staining

In brief, the samples fastened in 4% paraformaldehyde in phosphate‐buffered saline (PBS) (pH 7.4) were prepared. Subsequently, samples were nurtured for 10 min with PBS containing 0.25% Triton X‐100 after washing twice with ice‐cold PBS. Next, samples were washed in PBS three times for 5 min again. Next, 5% goat serum was added to cells to decrease nonspecific background for 30 min at room temperature. After that, samples were incubated at 4°C for 18 h with anti‐AQP4 antibody (rabbit, 1:100, bs‐0634R, Bioss), Tuj1 (mouse, 1:100, 2326315, Millipore), and then washed three times in 0.01 mol/L PBS for 5 min. Next, the secondary antibodies of Alexa Fluor594‐Conjugated AffiniPure (goat anti‐mouse immunoglobulin G, ZSGB‐BIO, ZF‐0512, 1:100, for Tuj1) and Dylight 488 (goat anti‐rabbit immunoglobulin G, Abbkine, A23220, 1:100, for AQP4) were used to incubate for 2 h at room temperature in darkness, followed by another three times washing with 0.01 mol/L PBS for 5 min. Lastly, samples were counterstained with DAPI for nuclei for 1 min, then rinsed with PBS slightly. Then, cells were observed and imaged under a fluorescent microscope (T1‐SM; Nikon). Average cell variety, somatic cell space, and extent of neurite in five random fields for each sample were analyzed by Image‐Pro 6.0 (MediaCybernetics).

### 2.15 Statistical analysis

All data were presented by mean ± standard deviation (SD). Student's *t* test was utilized for the contrast between two groups, while comparison among three or more independent groups was analyzed by one‐way ANOVA. The Kruskal–Wallis test was used for multiple cluster comparisons that failed to change to traditional homogeneity and homogeneity of variance. All the data were analyzed by SPSS version 21.0 (IBM Corporation). *p* < 0.05 was considered statistically significant.

## 3 RESULTS

### 3.1 HI induced the increase in water content

**Figure 1:**
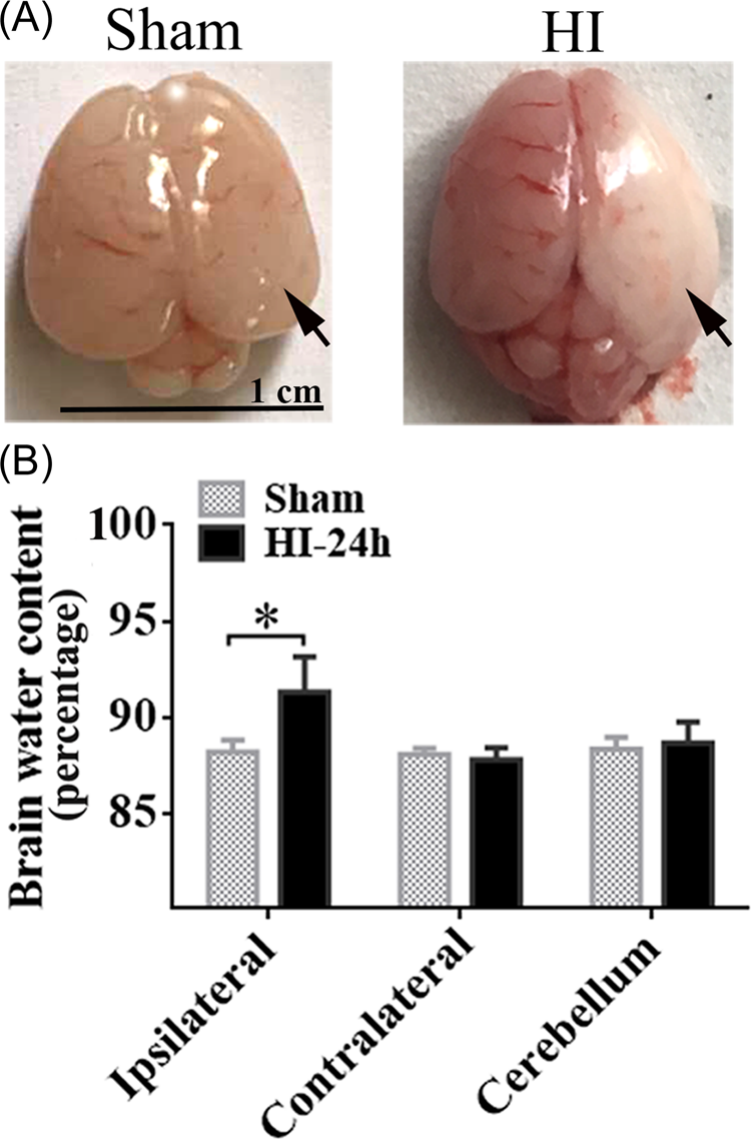
The changes in brain water content after HI. (A) Representing images of rat brains in the sham and HI groups. Black arrow stands for the infarction and edema. Scale bar = 1 cm. (B) The quantification of the brain water content of sham and HI groups in the ipsilateral, contralateral hemisphere, and cerebellum at 24 h after HI. HI, hypoxic‐ischemic. Data were presented as the mean± SD. *p < 0.05, *n* = 8/group. [Color figure can be viewed at wileyonlinelibrary.com]

To evaluate the details of NHIE pathogenesis, we successfully established infant HI rat models. Twenty‐four h after HI, whole brains were harvested to evaluate the change in brain water content. We found there was obvious cerebral edema and infarction in the ipsilateral hemisphere in the HI group (Figure 1A). Using wet and dry weight analysis, we further observed that the brain water content of the ipsilateral hemisphere in the HI group was notably increased as compared with that of the sham group (Figure 1B, *p* < 0.05), whereas there was no significant distinction in contralateral hemisphere and cerebellum (Figure 1B, *p* > 0.05).

### 3.2 Expression of AQP4 was appreciably increased in the cortex, hippocampus, and lung tissues after HI

**Figure 2:**
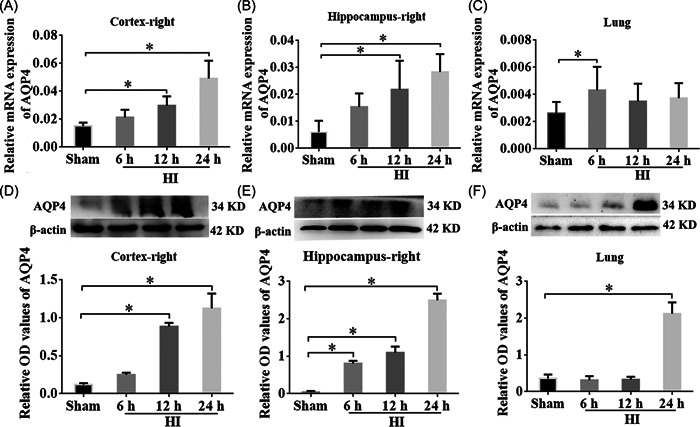
AQP4 was upregulated in the brain cortex, hippocampus, and lung after HI. The relative mRNA expression of AQP4 in (A) right cortex, (B) right hippocampus, and (C) lung of rats between the sham group and HI group at 6, 12, and 24 h post‐HI, respectively. Western blot stripes and proportional OD value quantification of AQP4 in (D) right cortex, (E) right hippocampus, and (F) lung of rats between the sham control and HI at 6, 12, and 24 h post‐HI, respectively. β‐actin served as an interior control. HI, hypoxia‐ischemia; OD, optical density. Data were presented as the mean ± SD. *p < 0.05, *n* = 8/group.

To explore the role of AQP4 in rats with NHIE, the relative mRNA expression of AQP4 within the right cortex, hippocampus, and lung tissues were examined using RT‐qPCR and WB at different time points (6 h, 12 h, 24 h post HI). The results showed that the mRNA expression levels of AQP4 were considerably elevated in the right cortex of the HI group compared with the sham group at 12 h and 24 h post‐HI and peaked at 24 h (Figure 2A, *p* < 0.05). Similar to the right cortex, the AQP4 mRNA level in the right hippocampus was also increased in the HI group at 12 h and 24 h after HI (Figure 2B, *p* < 0.05). Interestingly, the expression of AQP4 was substantially higher at 6 h post‐HI in the lungs than that in the sham group (Figure 2C, *p* < 0.05). Consistent with the RT‐qPCR results, WB data showed elevation of AQP4 proteins within the right cortex and hippocampus at 12 and 24 h after HI in comparison with the sham group (Figure 2D,E, *p* < 0.05). Although the mRNA of AQP4 was upregulated at 6 h post‐HI in the lung tissues, the protein levels were not elevated until 24 h post‐HI (Figure 2F, *p* < 0.05). These results specified that the expression of AQP4 was markedly raised after NHIE in both the ipsilateral cortex and hippocampus, as well as in lung tissues.

### 3.3 AQP4 was upregulated in Tuj1^+^ neurons under OGD condition

**Figure 3:**
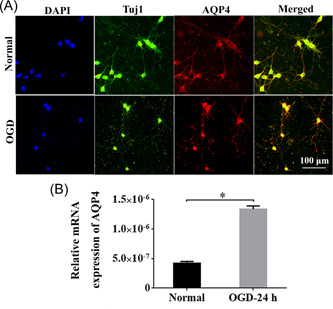
Impaired neurite growth and upregulated AQP4 expression in OGD neurons. (A) The immunostaining of Tuj1 and AQP4 under the normal and OGD conditions. (B) The mRNA levels of AQP4 in OGD and normal neurons. OGD, oxygen‐glucose deprivation; OGD‐24 h, 24 h after OGD. Data were presented as the mean ± SD.*p < 0.05. Scale bar = 100 μm, *n* = 3/group. [Color figure can be viewed at wileyonlinelibrary.com]

To pinpoint whether AQP4 was expressed in neurons, we isolated primary cortical neurons from rats. The dual immunostaining of Tuj1 and AQP4 revealed that the neurite outgrowth was obviously depressed in OGD neurons, and AQP4 was positive in Tuj1^+^ cortical neurons under both normal and OGD situations (Figure 3A). The quantification results revealed that the expression of AQP4 at 24 h post‐OGD was significantly higher than that of the normal group (Figure 3B, *p* < 0.05).

### 3.4 Effective siRNA fragment was screened and validated successfully

**Figure 4:**
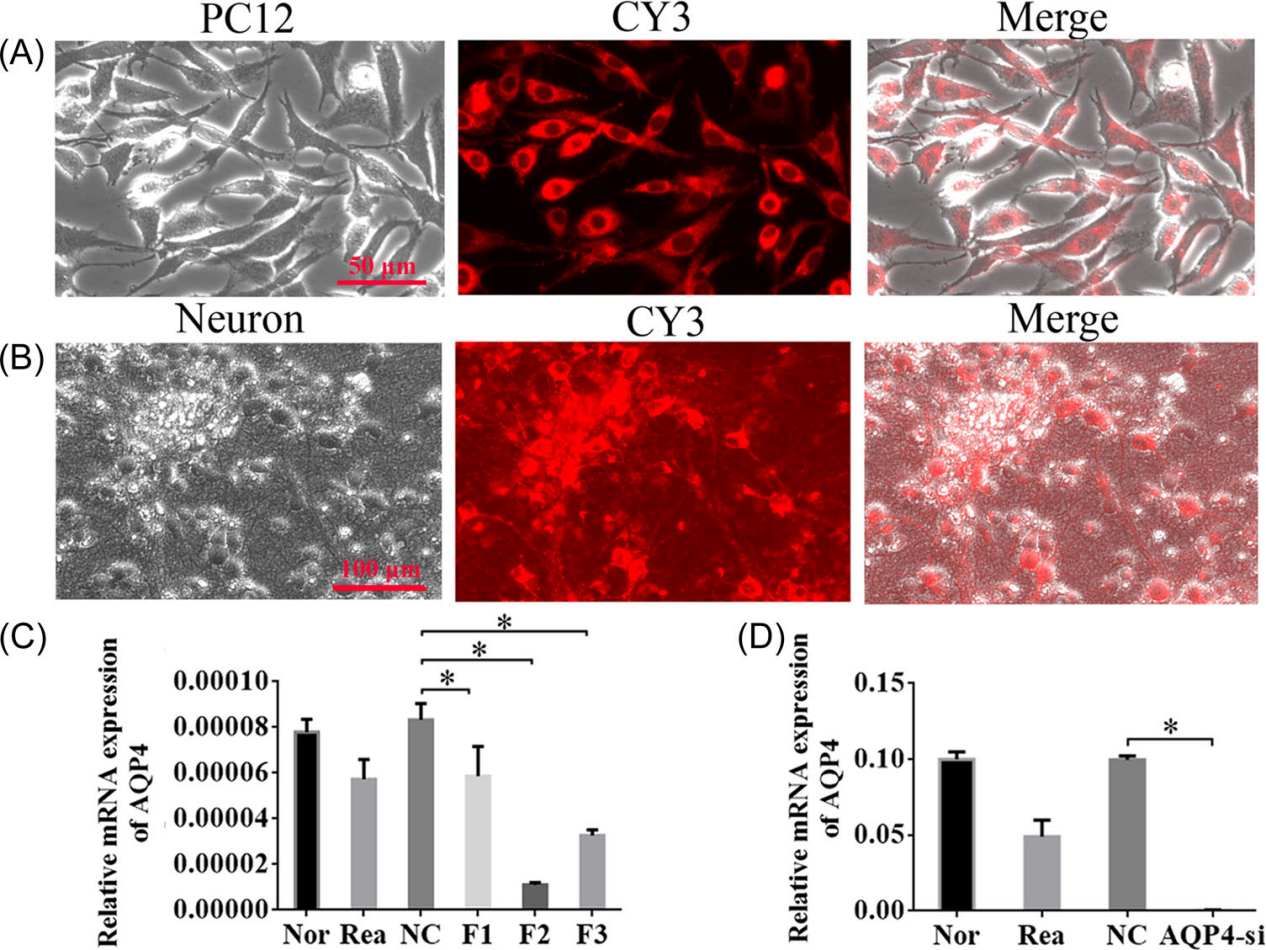
Identification of siRNA generating effective knockdown of AQP4. (A) Representative images of the PC12 cells cotransfected with CY3/siRNA (scale bar = 50 μm) and (B) isolated cortical neurons cotransfected with CY3/siRNA (scale bar = 100 μm). (C) RT‐qPCR analysis for AQP4 mRNA levels among Nor, Rea, NC, F1, F2, and F3 groups in PC12 cells, showing effective knockdown of AQP4 by F2. (D) AQP4 was successfully knocked down in isolated primary cortical neurons. AQP4‐si, silencing of AQP4; F1, No. 1 siRNA fragment; F2, No. 2 siRNA fragment; F3, No. 3 siRNA fragment; Nor, normal; OGD, oxygen‐glucose deprivation; NC, negative control group; Rea, reagent. Data were shown as the means ± SD. *p < 0.05. *n* = 3/group. [Color figure can be viewed at wileyonlinelibrary.com]

To confirm the role of AQP4 in vitro, we evaluated the impacts of the examinee series: F1‐F3, on the shushing work of AQP4. First, we tested the knockdown efficiency of candidate fragments in PC12 cells. Expression of CY3 showed successful cotransfection of CY3/siRNA (Figure 4A). Next, we analyzed these three siRNAs, F1‐F3 in isolated primary cortical neurons, which could also be successfully transfected by CY3/siRNA (Figure 4B). Among the three siRNAs, F2 showed the best efficiency for AQP4 knockdown in comparison with the other two (Figure 4C, *p* < 0.05). Therefore, F2 was selected for future experiments. As expected, a significant decrease in the expression of AQP4 was observed in AQP4 knockdown (F2 interference fragment, AQP4‐si) neurons compared to the NC group (Figure 4D, *p* < 0.05).

### 3.5 Interfering AQP4 promoted neurite outgrowth in OGD neurons

**Figure 5:**
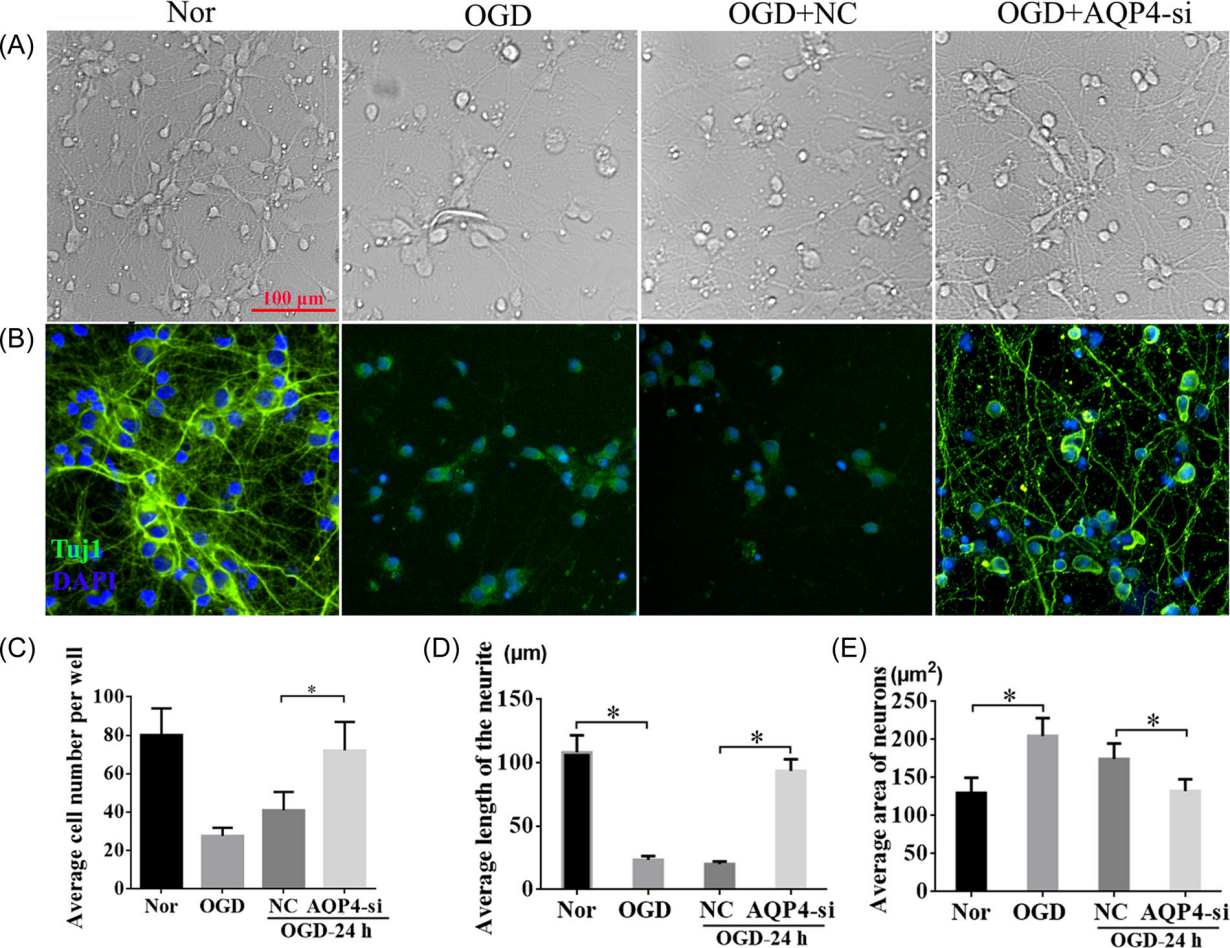
Silencing AQP4 promoted neuron survival and restored the neurite length after OGD. The morphology of neurons in the Nor, OGD, OGD + NC, and OGD + AQP4‐si groups were shown by (A) the light microscope and by (B) immunofluorescent costaining with Tuj1. Green fluorescence stands for the Tuj1 positive cells, and the nucleus was marked by blue fluorescence. The bar charts of (C) average cell number, (D) average length of neurites, and (E) average area of neurons. AQP4‐si, silencing of AQP4; NC, negative control group; Nor, normal; OGD, oxygen‐glucose deprivation. Data were presented as the mean ± SD. *p < 0.05. Scale bar = 100 μm, *n* = 3/group. [Color figure can be viewed at wileyonlinelibrary.com]

To test how AQP4 could affect neurons under the OGD condition, we examined Tuj1 expression among the normal, OGD, OGD + NC, and OGD + AQP4‐si groups (Figure 5A,B). These results showed that the neurons were broken clearly after OGD insult, whereas interference of AQP4 attenuated the nerve cell injury and promoted neurite outgrowth compared with the NC group (Figure 5A). The results of immunofluorescent staining of Tuj1 further confirmed the results (Figure 5B). Meanwhile, quantitative analysis revealed that the number of cells (Figure 5C), and the length of the neurite (Figure 5D) in the OGD group were considerably decreased compared with those within the normal group at 24 h after OGD, respectively. Conversely, after interfering with AQP4, cell numbers and the neurite length were restored to an extent similar to normal conditions without OGD, much higher than those in OGD + NC at 24 h post‐OGD (Figure 5C,D, *p* < 0.05). Furthermore, the area of neurons was notably increased after OGD, while AQP4‐si could markedly reduce the neuron area compared with that in the OGD + NC group, which indicated that interference of AQP4 could effectively ameliorate cell edema induced by OGD (Figure 5E, *p* < 0.05). These findings further confirmed that interference of AQP4 plays a crucial role in neuroprotection after OGD.

### 3.6 Long‐term neurological dysfunction induced by NHIE was alleviated after AQP4 knockout

**Figure 6:**
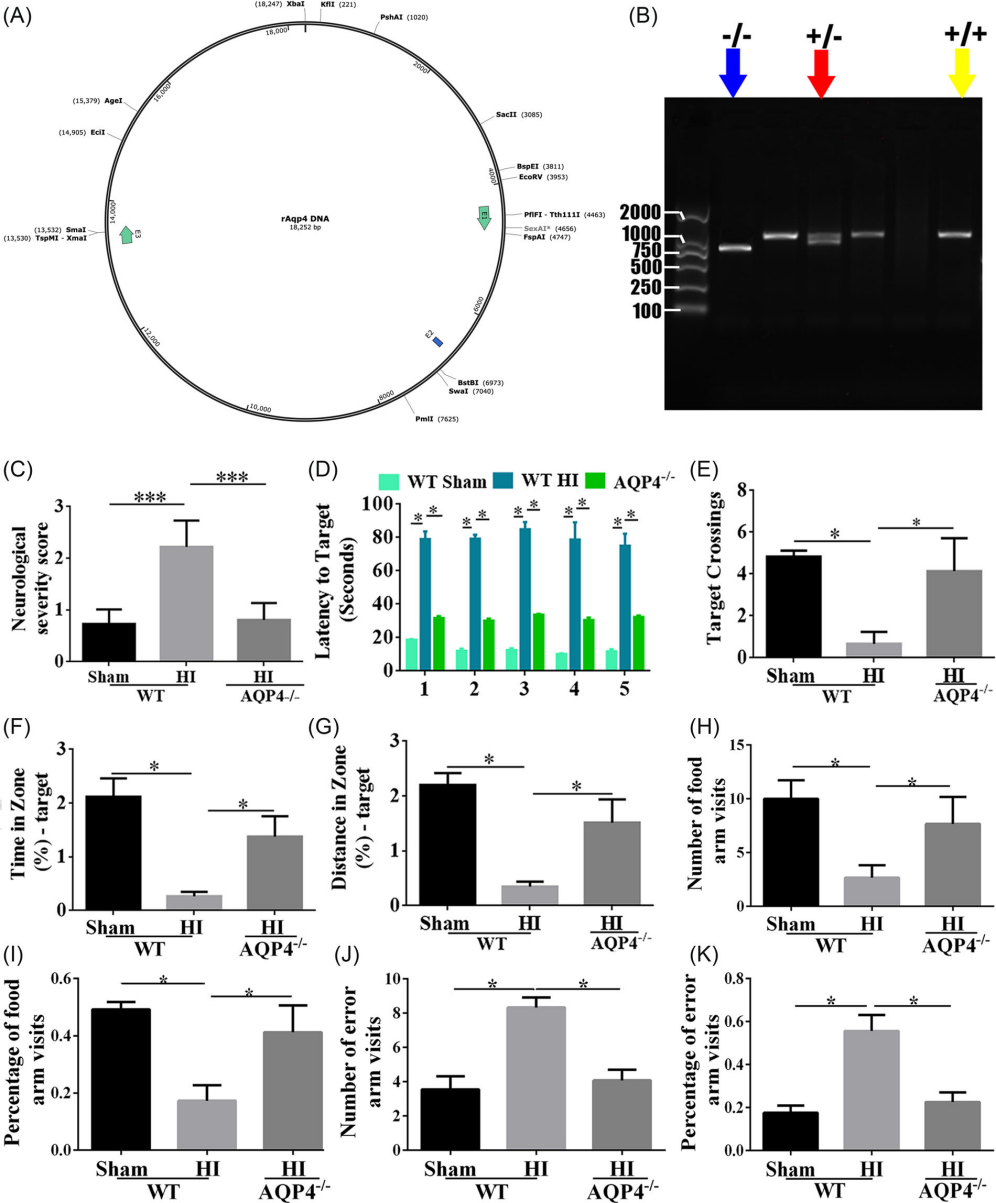
HI‐induced defected neurobehavioral function of rats was alleviated by AQP4 knockout. (A) Schematic construction of CRISPR/CAS9 knocking out AQP4 in rats. (B) Representative gel electrophoresis images of genotyping. Blue arrow represents homozygote; red arrow represents heterozygote; yellow arrow represents a wild type. (C) NSS score of sham‐WT, HI‐WT, and HI‐AQP4^−/−^ rats 1 month after HI. (D) The latency to target in the sham‐WT, HI‐WT, and HI‐AQP4^−/−^ groups within 5 days of training. (E) The number of target platform crossing, (F) the time, and (G) distance for traversing the first place of quadrant among these groups on the 6th day. (H) The number and (I) the percentage of food arm visits; (J) the number and (K) the percentage of error arm visits in the Y‐maze test among the rats in sham‐WT, HI‐WT, and HI‐AQP4^−/−^ groups. bp, base pair; HI‐WT, wild‐type rats with hypoxia‐ischemia; HI‐AQP4^−/−^, AQP4 knockout rats with hypoxia‐ischemia; KO, knockout; NSS, Neurological Severity Score; sham‐WT, wild‐type rats with the sham operation. Data are presented as the mean ± SD. *p < 0.05, ***p < 0.001, *n* = 8/group. [Color figure can be viewed at wileyonlinelibrary.com]

According to the in vitro results that AQP4‐si promoted neuron survival and neurite outgrowth, we then constructed AQP4^−/−^ rats by CRISPR/CAS9 (Figure 6A) to detect the function of AQP4 in HI‐induced long‐time neurological dysfunction in vivo. Wild type (WT) and knockout (KO) rats were utilized for the following experiments (examples of genotype are shown in Figure 6B). To assess the neurological function in the WT and AQP4 KO rats, an NSS score was performed 1 month after HI. Compared with the WT‐sham group, the severity of neurological deficit was considerably increased in WT‐HI rats (Figure 6C, *p* < 0.001). Whereas, AQP4 KO rats exhibited lower scores than WT‐HI rats, which was similar to WT‐sham rats (Figure 6C, *p* < 0.001). Additionally, a water maze test was performed to evaluate learning, memory and spatial orientation, and cognitive ability 1 month after HI. The WT rats subjected to HI spent more time finding the original location of the platform than WT‐sham rats (Figure 6D, *p* < 0.05). As expected, AQP4 KO rats performed better in the water maze than WT‐HI rats (Figure 6D, *p* < 0.05). Moreover, compared with WT‐HI rats, AQP4 KO rats had additional crossings over the initial location of the platform (Figure 6E, *p* < 0.05). Furthermore, the time (Figure 6F) and distance (Figure 6G) for finding the first location of the quadrant had a similar tendency with the target crossings among WT‐sham, WT‐HI, and AQP4 KO rats (*p* < 0.05). Similarly, the Y‐maze was performed to test the educational and memory abilities of rats after HI, which demonstrated that WT‐HI rats spent shorter time within the food arm, fewer numbers (Figure 6H, *p* < 0.05) and percentage (Figure 6I, *p* < 0.05) of food arm visits, but longer time in the error arm, and more error arm visits (Figure 6J,K, *p* < 0.05) than those in sham ones. However, the AQP4 KO rats spent more time in the food arm and less time in the error arm compared to WT‐HI rats. These results indicated that AQP4 knockout could alleviate HI‐induced long‐term learning and memory dysfunctions post HI.

### 3.7 Loss of AQP4 resulted in elevated expression levels of GAP43 in OGD neurons and HI rats

**Figure 7:**
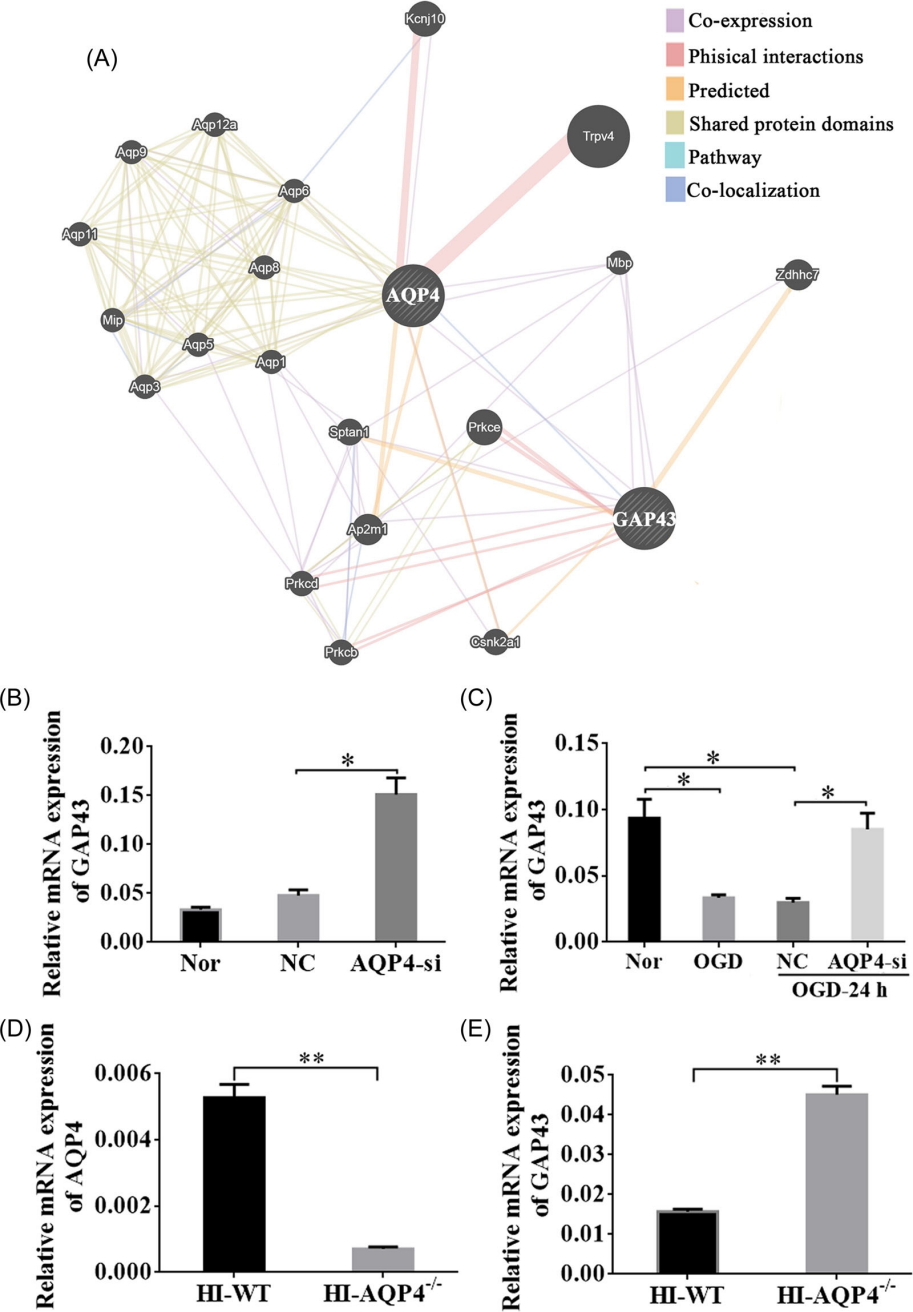
AQP4 loss resulted in upregulation of GAP43 in OGD neurons and HI rats. (A) Gene network diagram of AQP4 and GAP43 by GeneMANIA analysis. The purple line represents co‐expression relation; the blue line represents pathway relation. (B) Quantitative relative expression of GAP43 in Nor, NC, and AQP4‐si groups under normal conditions, and (C) in the groups of Nor, OGD, NC, and AQP4‐si at OGD‐24 h under OGD condition (*n* = 3/group). (D) The relative mRNA expression of AQP4 between HI‐WT and HI‐AQP4^−/−^ groups. (E) The relative mRNA expression of GAP43 between HI‐WT and HI‐AQP4^−/−^ groups (*n* = 8/group). AQP4‐si, silencing of AQP4; HI‐AQP4^−/−^, AQP4 knockout rats with hypoxia‐ischemia; HI‐WT, wild‐type rats with hypoxia‐ischemia; NC, negative control group; Nor, normal; OGD, oxygen‐glucose deprivation; OGD‐24 h, 24 h after OGD. Data are presented as the mean ± SD. *p < 0.05, **p < 0.01. [Color figure can be viewed at wileyonlinelibrary.com]

To explore the potential mechanism of AQP4 in the regulation of neurite length, we performed a GeneMANIA analysis to determine the coexpression relationship of AQP4 with other molecules (Figure 7A). We found that GAP43 greatly interacted with AQP4 (Figure 7A). Therefore, RT‐qPCR was carried out to confirm their regulating relation. Quantitative analysis disclosed that the relative expression of GAP43 was redoubled after interfering with AQP4 compared with the NC group under normal conditions (Figure 7B, *p* < 0.05). Moreover, we found that after OGD, the expression level of GAP43 was decreased than that in normal conditions (Figure 7C, *p* < 0.05). However, interference of AQP4 could obviously upregulate the expression of GAP43 at 24 h post‐OGD (Figure 7C, *p* < 0.05). Furthermore, WT and AQP4^−/−^ rats were also employed to verify this relationship, which revealed that relative mRNA expression of AQP4 was markedly decreased in AQP4^−/−^ rats as compared with WT‐HI rats (Figure 7D, *p* < 0.005), whereas the relative mRNA expression of GAP43 was clearly accrued in AQP4^−/−^ groups after HI (Figure 7E, *p* < 0.005). These results disclosed that downregulation of AQP4 could upregulate the expression of GAP43 so as to exert neuroprotective effects.

## 4 DISCUSSION

In our research, we utilized both in vivo and in vitro models to evaluate AQP4 in acute HIE and long‐term recovery after NHIE. Our results showed that AQP4 downregulation effectively improved neurite growth in the OGD neurons and long‐run learning and memory function in rats subjected to NHIE. In addition, the possible mechanism involved in axon regeneration and upregulation of growth‐associated protein‐43 (GAP43). Taken together, our findings provide a novel interpretation for AQP4 signaling as a potential therapeutic target in the treatment of NHIE.

### 4.1 P7 rats were selected for NHIE model establishment

In accordance with the previous literature, the P7 neonatal rats suffered from unilateral carotid artery occlusion to establish the NHIE model in this study. P7 rats were selected because the peak brain growth occurs for rats at this time point, which would appear at full‐term in humans.^31^, ^32^ In addition, this is the time when cortical myelination for rodents just starts, equivalent to 34 weeks of human pregnancy.^33^ In the present study, P7 neonatal rats were chosen for ligation of the correct arteria carotid artery following 2 h hypoxia. Afterward, the cerebral damages were principally triggered within the right hemisphere. Therefore, we tend to principally use the anemia ipsilateral brain (right cerebral hemisphere) within the later detection.^31^

### 4.2 Downregulation of AQP4 improved axon regrowth and long‐term neural behavior after HIE

In the present study, interference of AQP4 or AQP4^−/−^ rats was performed to understand the role of AQP4 in acute and semipermanent neurological impairments of HIE. Our data found that neurite outgrowth was more obvious after silencing AQP4 in the OGD model in vitro. Meanwhile, AQP4^−/−^ could notably improve neurobehavioral impairment after HIE in vivo, which was reflected by the check of NSS score, water maze, and Y‐maze 1 month after HIE. We speculate that increased neurite outgrowth is secondary to the primary preservation of neurons, allowing greater regeneration. Accumulating evidence had indicated that the amount of AQP4 expression was altered after cerebral ischemia. In Yang's study, they applied intracerebral injection of the plasmid containing AQP4 siRNA in neonatal piglets to knock down AQP4 expression in vivo, and observed changes in brain edema and neurological functions after AQP4 knockdown.^34^ In the study with permanent middle cerebral artery occlusion (MCAO) in rats, the expression of AQP4 mRNA and protein was increased.^35^ However, hypoxia significantly decreased expression levels of AQP4 in cultured rat astrocytes.^36^ Another study suggested that AQP4 induction in astrocyte end‐feet limits edema formation by facilitating water clearance from/to the vascular compartment.^37^ In addition, after temporary MCAO, the striatal core displayed a loss of perivascular AQP4 with no sign of resulting recovery, and the cortical border had no loss of perivascular AQP4.^38^ In our research, we dynamically determined the change of expression levels of AQP4 at 6, 12, and 24 h after HI, and the data showed significantly increased level of AQP4 in both cortex and hippocampus.

For the function of AQP4, it was reported that AQP4 played a critical part in controlling water fluxes into and out of the brain parenchyma. Likewise, AQP4 also contributes to the formation and/or the absorption of the brain lump resulting from cerebral ischemia.^39^ Numerous studies have proved that AQP4 downregulation in reactive astrocytosis attenuates brain edema after focal cerebral ischemia.^40^ In addition, AQP4 is coupled to K equilibrium in vivo. The downregulation of outward potassium (K^+^) conductance during HI may prevent the emission of intracellularly accumulated K^+^ ions, thus resulting in osmotically derived water influx into astrocytes via AQP4 and then cell swelling.^41^, ^42^ Additionally, several studies have shown that erythropoietin and IPostC could reduce brain edema and cell swelling might be mediated by inhibiting AQP4 in neonatal HI rats in the short term.^26^, ^43^ In our study, we found AQP4 could co‐express with Tuj1 staining and also has functional effects on neurons, thereby participating in the process of brain edema and axonal growth in HIE disease.

The present study identified the novel function of downregulation of AQP4 in the axonal growth in OGD neurons. Interference of AQP4 could promote the growth of neurites and increase survival in neurons after OGD. Furthermore, NHIE rats with AQP4^−/−^ exhibited better long‐term neurobehavioral outcomes. Thus, our results indicated that downregulation of AQP4 was related to axon regrowth and could promote the recovery of long‐term neurological dysfunction elicited by NHIE; in addition, AQP4 could not only function via astrocytes but also through neurons.

### 4.3 Upregulated GAP43 might be involved in the mechanism of the axon growth after the interference of AQP4 in the NHIE‐induced brain damage

We found GAP43 was closely correlated with AQP4 in both pathway and co‐expression channels through GeneMANIA prediction. RT‐qPCR verification indicated that interference of AQP4 could markedly increase the expression of GAP43 and promote neurite growth. It has been known that GAP43 was originally found in neurons and also the expression is especially high throughout nerve fiber growth and through development and regeneration in each central and peripheral nervous system.^44^ However, the expression diminishes with a decrease in nerve fiber arborization and synaptogenesis and remains solely in high‐plasticity areas like the hippocampus and eventually the neural structure in mice.^45^ This supermolecule is strictly preserved among vertebrates, indicating that it plays a necessary role.^46^

Some studies found that overexpression of GAP43 promoted nerve growth in transgenic mice and method outgrowth in civilized cells.^47^, ^48^ In addition, proof within the past few years showed that it should be helpful to act GAP43 as an associate in nursing adapter macromolecule due to the association and potential binding of GAP43 with a variety of various molecules, together with PKC, PIP2, actin, synaptophysin, and so forth.^49^, ^50^, ^51^, ^52^ However, there was no study to show the association with AQP4. Our data demonstrated that downregulation of AQP4 could promote neurite regrowth after OGD. Furthermore, PCR verified that the expression of GAP43 was notably higher in the condition of interfering AQP4 at 24 h after OGD. Thus, these results revealed that the downregulation of AQP4 promoting neurite outgrowth in NHIE might be associated with the upregulation of GAP43.

Taken together, silencing AQP4 could enhance axon regeneration and ameliorate the long‐term neurobehavioral recovery after HIE, and the underlying mechanism was related to the upregulation of GAP43. Our research provides a vital explanation for the therapeutic effects of AQP4 silencing in neurological function recovery from HIE, and it may underlie the feasible foundation for the treatment of HIE in future medical experiments, and provide a basis for the development of new drugs based on AQP4. Nevertheless, limitations exist in this study since we have not detected changes in other vital indicators as a result of the knockout of AQP4 except GAP43. More comprehensive investigations on AQP4‐related mechanisms in NHIE are necessary for later studies.

## AUTHOR CONTRIBUTIONS

Belegu Visar and Li Chen were accountable for the study design, the guidance of the study, as well as the revision of the paper. Qi‐Qin Dan, Ya‐Xin Tan, and Zheng Ma participated in behavioral detection, molecular and histological experiments, and data analysis. All authors have read and approved the final version of the manuscript.

## CONFLICT OF INTEREST

Professor Belegu Visar is a member of Ibrain Journal editorial board and is not involved in the peer review process of this article.

## ETHICS STATEMENT

All the trials were disbursed in conformity to the care and use of laboratory animals publicized by the Ministry of Science and Technology of the People's Republic of China and approved by the Animal Care and Use Committee of Kunming Medical University (kmmu2019039) and were in compliance with the National Institutes of Health Guide for the Care and Use of Laboratory Animals.

## Data Availability

The data generated throughout the present study are obtainable from the corresponding author on request.
